# PRMT5 promotes epithelial‐mesenchymal transition via EGFR‐β‐catenin axis in pancreatic cancer cells

**DOI:** 10.1111/jcmm.14894

**Published:** 2019-12-18

**Authors:** Lu Ge, Huizhi Wang, Xiao Xu, Zhengrong Zhou, Junbo He, Wanxin Peng, Fengyi Du, Youli Zhang, Aihua Gong, Min Xu

**Affiliations:** ^1^ Department of Gastroenterology Affiliated Hospital of Jiangsu University Zhenjiang China; ^2^ Department of Gastroenterology Danyang People's Hospital Zhenjiang China; ^3^ Department of Cell Biology School of Medicine Jiangsu University Zhenjiang China

**Keywords:** AKT, EGFR, epithelial‐mesenchymal transition, GSK3β, protein arginine methyltransferase 5, β‐catenin

## Abstract

Protein arginine methyltransferase 5 (PRMT5) has been implicated in the development and progression of human cancers. However, few studies reveal its role in epithelial‐mesenchymal transition (EMT) of pancreatic cancer cells. In this study, we find that PRMT5 is up‐regulated in pancreatic cancer, and promotes proliferation, migration and invasion in pancreatic cancer cells, and promotes tumorigenesis. Silencing PRMT5 induces epithelial marker E‐cadherin expression and down‐regulates expression of mesenchymal markers including Vimentin, collagen I and β‐catenin in PaTu8988 and SW1990 cells, whereas ectopic PRMT5 re‐expression partially reverses these changes, indicating that PRMT5 promotes EMT in pancreatic cancer. More importantly, we find that PRMT5 knockdown decreases the phosphorylation level of EGFR at Y1068 and Y1172 and its downstream p‐AKT and p‐GSK3β, and then results in down‐regulation of β‐catenin. Expectedly, ectopic PRMT5 re‐expression also reverses the above changes. It is suggested that PRMT5 promotes EMT probably via EGFR/AKT/β‐catenin pathway. Taken together, our study demonstrates that PRMT5 plays oncogenic roles in the growth of pancreatic cancer cell and provides a potential candidate for pancreatic cancer treatment.

## 1 INTRODUCTION

Pancreatic cancer is the most common malignancy of the pancreas, with a dismal 5‐year survival rate of less than 5% and a median survival of <11 months. This dismal outcome can be attributed to the lack of early diagnoses and effective interventions. Additionally, conventional treatment approaches such as surgery, chemotherapy and radiation have generally had little impact on the course of this aggressive cancer despite efforts over the past several years.^1^ Therefore, the detection and diagnosis of pancreatic cancer in the early stage are extremely urgent.

Emerging evidence has demonstrated that epithelial‐mesenchymal transition (EMT) plays an essential role in the progression of pancreatic cancer.^2^ It is a biologic process in which epithelial cells transform into special cells with mesenchymal phenotypes, resulting in enhanced invasion and metastasis. Concomitantly, epithelial markers such as E‐cadherin are down‐regulated, whereas mesenchymal markers including Vimentin, collagen I and β‐catenin are up‐regulated.^3^ In 2019, a study provided evidence for the positive effects of SLC34A2 on EMT phenotype in glioma cell lines via the EGFR/PI3K/AKT signalling.^4^ However, the molecular mechanisms that act upstream of these factors in various physiological and pathologic contexts in pancreatic cancer are not well characterized. Therefore, it is necessary to discover the specific mechanism in pancreatic cancer to provide novel prognostic and treatment targets.

Protein arginine methyltransferases (PRMTs) plays critical roles in a variety of cellular processes including transcriptional regulation, chromatin regulation, signal transduction, RNA processing and DNA damage repair.^5^ PRMT5, the type II protein arginine transferase, catalyses the symmetrical dimethylation of arginine residues on histone and non‐histone substrates and plays multiple roles in cellular processes, including differentiation, proliferation, apoptosis and ribosome biogenesis.^6^ Furthermore, several studies have shown that PRMT5 plays an important role in the development and progression of human cancers including glioblastoma,^7^ colorectal cancer,^8^ breast cancer,^9^ lymphoma,^10^ prostate cancer 11 and lung cancer.^12^ Recently, Menin and PRMT5 were found to suppress Glucagon‐like‐peptide‐1 receptor (GLP1R) transcription to inhibit the proliferation of β‐cell in pancreatic diseases.^13^ In addition, PRMT5 was proved to inhibit the tumour suppressor FBW7, resulting in increasing c‐Myc levels to promote the proliferation of and aerobic glycolysis in pancreatic cancer cells.^14^ By far, there are not enough studies uncovering the roles of PRMT5 about EMT in pancreatic cancer.

In this study, we examined the roles of PRMT5 in pancreatic cancer and elucidated the underlying mechanism. Our data showed that PRMT5 promoted cell proliferation, migration and invasion in pancreatic cancer cells, and promoted tumorigenesis. Importantly, PRMT5 promoted EMT probably via EGFR/AKT/β‐catenin pathway.

## 2 MATERIALS AND METHODS

### 2.1 Cell lines and culture conditions

The pancreatic cancer cell lines PaTu8988 and SW1990 were obtained from ATCC (USA) and Cancer Cell Repository (Shanghai, China). Cells were maintained in Dulbecco's Modified Eagle's Medium (DMEM) with 10% foetal bovine serum (FBS) at standard cell culture conditions (37°C, 5% CO_2_ in humidified incubator). DMEM, FBS and trypsin were purchased from Gibco (Invitrogen).

### 2.2 Plasmids construction

The oligo sequence of sh‐PRMT5‐3′UTR included: PRMT5 shRNA (F): 5′‐CCGGGG CTCAAGCCACCAATCTATGCTCGAGCATAGATTGGTGGCTTGAGCCTTTTTG‐3′, PRMT5 shRNA (R): 5′‐AATTCAAAAAGGCTCAA GCCACCAATCTATGCTC GAGCATAGATTGGTGGCTTGAGCC‐3′. The PRMT5 shRNA sequence was inserted into the EcoR I and Age I site of the pLKO.1‐TRC plasmid and ligated into the vector (Sigma).

### 2.3 Lentivirus production and cell infection

The packaging plasmid psPA ×2 and the envelope plasmid pMD2.G were purchased from Sigma (MO, USA). PLKO.1‐sh‐PRMT5 vector was cotransfected with psPA ×2 and pMD2.G into HEK293T cells using Lipofectamine2000 (Invitrogen). Viruses were harvested 48 hours after transfection, and viral titres were determined. Cells were infected with 1 × 10^6^ recombinant lentivirus transduction units in the presence of 8 mg/mL polybrene (Sigma). Puromycin (1:10 000 dilutions) was added to cells until the cells in blank group died off.

### 2.4 Transient transfection

The plasmids pHA‐venus and pHA‐PRMT5 were kind gifts from Professor Mo. Cells were plated in six‐well plates at a density of 4 × 10^5^ cells/well. After 24 hours of culture, the medium was replaced by Opti‐MEM (Invitrogen) and cultured. In total, 2 μg plasmid was transfected using 6 μL Lipofectamine^®^ 2000 Transfection Reagent. After incubation for another 48 hours, the treated cells were determined using Western blot analysis, transwell or cell counting Kit‐8 assay.

### 2.5 Western blot

Whole‐cell lysates were prepared and Western blot was carried out as recently described.^15^ The following antibodies were used: PRMT5, β‐tubulin, collagen I (Abcam), E‐cadherin, Vimentin, β‐catenin, p‐EGFR (Y1068), EGFR, (Cell Signaling Technology), Akt, p‐Akt, GSK‐3β, p‐GSK‐3β, p‐EGFR (Y1172), HA (ImmunoWay) and HRP‐conjugated secondary antibodies (Pierce).

### 2.6 Cell counting kit‐8 assay

The measurement of viable cell mass was performed with Cell Counting Kit‐8 (Promega) according to manufacturer's instructions. Briefly, 3000 cells/well were seeded in a 96‐well plate, grown in an incubator (5% CO_2_, 37°C). Respectively in first day, second day, third day and fourth day, 10 μL CCK‐8 was added to each well, cells were incubated at 37°C for 2 hours, and the absorbance was finally determined at 490 nm.

### 2.7 Colony formation assay

Cells were seeded in six‐well plates at a density of 1000 cells per well and cultured in incubator (5% CO_2_, 37°C) for two weeks. At the end of the incubation, the cells were fixed with 4% paraformaldehyde, stained with 0.1% crystal violet. Megascopic cell colonies were counted using Image‐Pro Plus 5.0 software (Media Cybernetics). Colony formation rate = (number of colonies/number of seeded cells) × 100%. Each measurement was performed in triplicate, and the experiments were each conducted at least three times.

### 2.8 Cell migration assay

A total of 24‐well inserts (8‐μm pore size) were purchased from BD Biosciences. 5 × 10^4^ PaTu8988 cells and 1 × 10^5^ SW1990 cells were seeded in serum‐free DMEM on the top of chambers. The lower chambers were filled with 500 μL DMEM supplemented with 10% foetal bovine serum. After incubation at 37°C (PaTu8988, 24 hours; SW1990, 36 hours), cells on the upper surface of the filter were removed with a cotton swab, while the invaded cells were fixed with 4% paraformaldehyde, stained with 0.1% crystal violet, photographed (×20) in five independent fields for each well and counted. Each test was repeated in triplicate.

### 2.9 Cell invasion assay

Cell invasion was determined using a Boyden chamber assay. 5 × 10^4^ PaTu8988 cells and 1 × 10^5^ SW1990 cells were seeded in serum‐free DMEM in the upper wells, which have already been covered with a layer of BD Matrigel Basement Membrane. The cells were later processed similar to that of cell migration assay, photographed (×20) in five independent fields for each well, and counted. Each test was repeated in triplicate.

### 2.10 Xenograft mouse model

The protocol was approved by the Institutional Animal Care and Use Committee of Jiangsu University, Zhenjiang, China. PaTu8988 and SW1990 cells (2.0 × 10^6^ cells/site) stably transfected with sh‐EGFP, sh‐PRMT5, pHA‐Venus and pHA‐PRMT5 were subcutaneously injected into 5‐week‐old BALB/c nude mice (Shanghai SLAC Laboratory Animal Co., Ltd) to generate xenografts. There are five female mice in each group. The tumour volume was measured every week after injection and calculated using the following formula: 4/3 πlength × width × height.

### 2.11 qRT‐PCR

Total RNA was extracted using RNAiso Plus (Takara). Reverse transcription was performed using RevertAid First Strand cDNA Synthesis Kit (Thermo, Waltham) according to the manufacturer's specification. Real‐time PCR was performed in triplicate in 20 μL reactions with iQ SYBR Premix Ex Taq Perfect Real Time (Bio‐Rad Laboratories, Inc), 50 ng first strand cDNA and 0.2 μg each primer. The primers for qRT‐PCR were in Table 1. Samples were cycled once at 95°C for 2 minutes and then subjected to 35 cycles of 95°C, 56°C and 72°C for 30 seconds each. The relative mRNA content was calculated using the 2^−ΔΔCt^ method with GAPDH as an endogenous control.

**Table 1:** DNA and RNA nucleotide sequences

PRMT5‐F	CTGTCTTCCATCCGCGTTTCA
PRMT5‐R	GCAGTAGGTCTGATCGTGTCTG
GAPDH‐F	TGGGGAAGGTGAAGGTCGG
GAPDH‐R	CTGGAAGATGGTGATGGGA
β‐catenin‐F	AAAGCGGCTGTTAGTCACTGG
β‐catenin‐R	CGAGTCATTGCATACTGTCCAT
Vimentin‐F	AGTCCACTGAGTACCGGAGAC
Vimentin‐R	CATTTCACGCATCTGGCGTTC
Collagen I‐F	GAGGGCCAAGACGAAGACATC
Collagen I‐R	CAGATCACGTCATCGCACAAC
E‐cadherin‐F	ATTTTTCCCTCGACACCCGAT
E‐cadherin‐R	TCCCAGGCGTAGACCAAGA
shPRMT5‐F	CCGGGG CTCAAGCCACCAATCTATGCTCGAGCATAGATTGGTGGCTTGAGCCTTTTTG
shPRMT5‐R	AATTCAAAAAGGCTCAA GCCACCAATCTATGCTCGAGCATAGATTGGTGGCTTGAGCC
sh‐EGFP‐F	CCGGTACAACAGCCACAACGTCTATCTCGAGATAGACGTTGTGGCTGTTGTATTTTTG
sh‐EGFP‐R	AATTCAAAAATACAACAGCCACAACGTCTATCTCGAGATAGACGTTGTGGCTGTTGTA

### 2.12 EGFR inhibitor

Cells were incubated in culture medium with 10% FBS supplemented with EGFR inhibitor (Erlotinib, CP‐358774, MCE) over the concentration range 0, 10 μmol/L.^16^ Cells were harvested at 72 hours after treatment.

### 2.13 Statistical analysis

All statistical analyses were carried out using the SPSS statistics software package. All data are presented as mean ± SD from at least three independent experiments. Comparisons between groups were analysed using the Student's *t* test (two groups) or an one‐way ANOVA (multiple groups). Kaplan‐Meier survival was analysed using log‐rank analysis. *P* < .05 was considered statistically significant.

## 3 RESULTS

### 3.1 PRMT5 is profiled in pancreatic cancers and different pancreatic cancer cells

**Figure 1:**
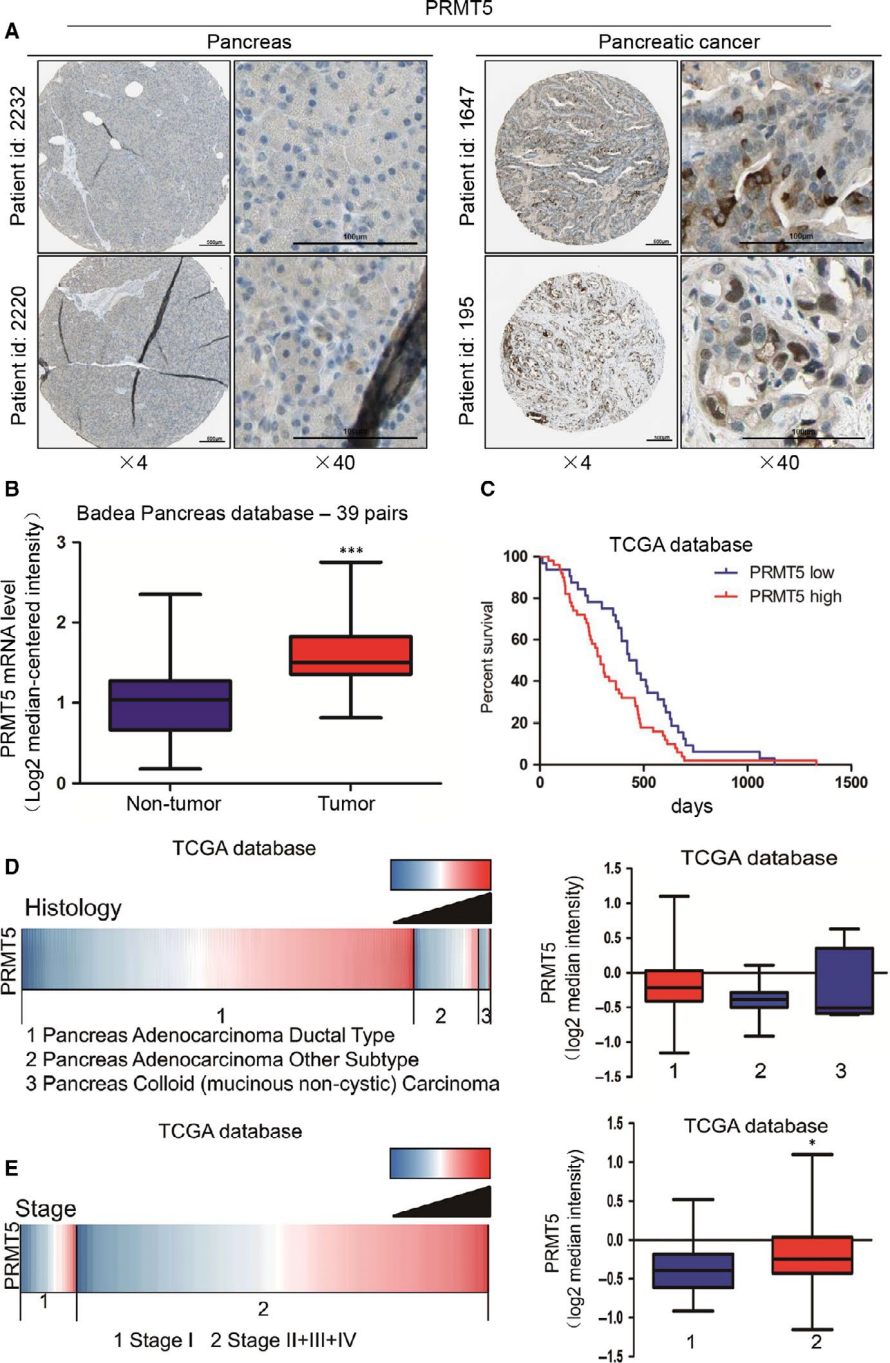
PRMT5 is profiled in pancreatic cancers and different pancreatic cancer cells. A, PRMT5 protein expression in pancreatic cancer tissues and normal pancreatic tissues was analysed through the human protein atlas (http://www.proteinatlas.org). Magnification, ×4; bars, 500 μm. Magnification, ×40; bars, 100 μm. B, Analysis of PRMT5 mRNA levels in 39 pairs of pancreatic cancer and non‐tumour tissues in Badea pancreas database. N = 39 for non‐tumour group, and N = 39 for tumour group. ***P < .001. C, Analysis of the TCGA database indicates that PRMT5 expression is correlated with patient’ overall survival. N = 32 for PRMT5‐low group, and N = 50 for PRMT5‐high group. *P* = .0345 was determined by log‐rank test. D, Analysis of the TCGA database indicates PRMT5 correlates with clinicopathological features. The results are presented by heat map (left panel) and box plot (right panel). N = 139 for pancreas adenocarcinoma ductal type group, N = 23 for pancreas adenocarcinoma other subtype group, and N = 4 for pancreas colloid (mucinous non‐cystic) carcinoma group. *P* = .0707 (E) Analysis of the TCGA database indicates PRMT5 is associated with stage in pancreatic cancer. The results are presented by heat map (left panel) and box plot (right panel). N = 20 for stage I group, N = 147 for stage II + III + IV group. *P < .05

To confirm the clinical relevance of PRMT5 expression, we first analysed the PRMT5 protein expression in clinical specimens from the human protein atlas (http://www.proteinatlas.org). We found that PRMT5 had the positive strong expression in pancreatic cancer and negative weak expression in normal pancreas (Figure 1A). Furthermore, we analysed the PRMT5 mRNA level in clinical specimens from oncomine (http://www.oncomine.org). We found that PRMT5 mRNA level was higher in pancreatic cancer tissues than that in normal pancreatic tissues (1.03 ± 0.48 vs 1.60 ± 0.39, *P* < .001, n = 78) in Badea pancreas database (Figure 1B). These results suggest that PRMT5 is up‐regulated in pancreatic cancer.

Subsequently, patients with high expression of PRMT5 had a median survival of 293 days as compared with 448 days for the patients with low expression of PRMT5 (HR = 0.6199, *P* < .05) (Figure 1C). Because of the limitations of the Badea pancreas database information, we investigated more information in TCGA database (https://genome-cancer.ucsc.edu) and evaluated the correlation of PRMT5 expression with clinicopathological features, tumour stage and patients’ outcome. PRMT5 expression in pancreas adenocarcinoma ductal was higher than that in pancreas adenocarcinoma other subtype and pancreas colloid (mucinous non‐cystic) carcinoma (Figure 1E). Moreover, further analysis showed that PRMT5 expression in stage I was lower than that in stage II + III + IV groups (Figure 1F).

### 3.2 PRMT5 promotes cell proliferation in pancreatic cancer cells and tumorigenesis

**Figure 2:**
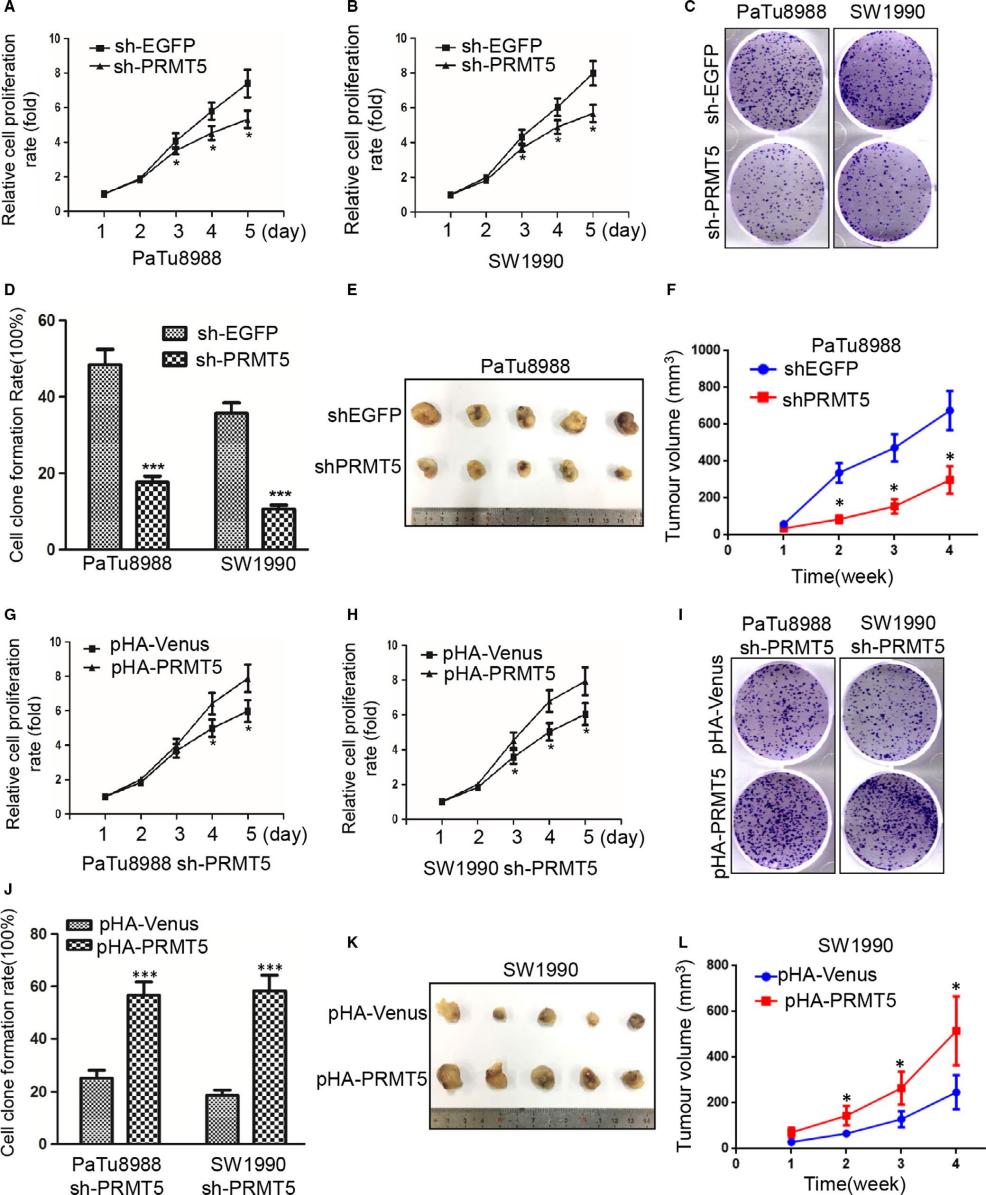
PRMT5 promotes cell proliferation in pancreatic cancer cells and tumorigenesis. A‐B, CCK‐8 assay showed that PRMT5 knockdown inhibited PaTu8988 and SW1990 cell growth rate (Student's *t* test:*P < .05). C‐D, Clone formation assays in PaTu8988 and SW1990 cells. PRMT5 knockdown inhibited cell clone formation (Student's *t* test:*P < .05). The number of clones with at least 50 cells per colony and strong, high dense staining was counted. The rates of colony formation were 48.33% and 17.67% in sh‐EGFP and sh‐PRMT5 PaTu8988 cells, and 35.67% and 10.67% in sh‐EGFP and sh‐PRMT5 SW1990 cells, respectively. E‐F, PaTu8988 cells with PRMT5 down‐regulation were injected (2.0 × 10^6^ cells/site) subcutaneously into a mice, and the tumour volume was measured weekly (n = 5 mice). *P < .05. G‐H, CCK‐8 assay showed that ectopic PRMT5 re‐expression in PaTu8988 and SW1990 sh‐PRMT5 stable infected cells promoted cell proliferation rate (Student's *t* test: *P < .05). I‐J, Clone formation assays in PaTu8988 and SW1990 sh‐PRMT5 stable infected cells. Ectopic PRMT5 re‐expression in PaTu8988 and SW1990 sh‐PRMT5 stable infected cells promoted cell clone formation (Student's *t* test: ***P < .001). The rates of number of colonies (defined as ≥50 cells) were 25.33% and 56.67% in PaTu8988 cells, 18.67% and 58.33% in SW1990 cells, respectively. K‐L, SW1990 cells with PRMT5 up‐regulation were injected (2.0 × 10^6^ cells/site) subcutaneously into a mice, and the tumour volume was measured weekly (n = 5 mice), *P < .05

To investigate the effects of PRMT5 on cell growth in pancreatic cancer cells, we first used the CCK8 assay to determine the growth curves and then evaluated their ability of colony formation. As showed in Figure 2A‐D, PRMT5 knockdown significantly inhibited the proliferation of pancreatic cancer cells and resulted in the smaller colonies and lower colony density compare to control in both PaTu8988 and SW1990 cells. It is suggested that PRMT5 is critical for the cell proliferation in pancreatic cancer cells.

To confirm the above results, the sh‐PRMT5 PaTu8988 cells or SW1990 cells were transfected with pHA‐Venus or pHA‐PRMT5 plasmids, respectively. Expectedly, PRMT5 promoted the cell proliferation and increased the colony sizes and densities in pHA‐PRMT5 groups compared with pHA‐venus groups (Figure 2G‐J), indicating that PRMT5 could rescue the inhibition of proliferation resulted from PRMT5 knockdown in PaTu8988 and SW1990 cells, indicating that ectopic PRMT5 re‐expression could rescue the effect of knockdown‐mediated inhibition on colony formation. Furthermore, tumour growth was inhibited in xenograft mouse model injected sh‐PRMT5 PaTu8988 cells while promoted in xenograft mouse model injected pHA‐PRMT5 SW1990 cells (Figure 2E‐L). It is indicated that PRMT5 also significantly increased tumour growth. It is further suggested that PRMT5 plays an important role in cell proliferation of pancreatic cancer cells.

### 3.3 PRMT5 promotes cell migration in pancreatic cancer cells

**Figure 3:**
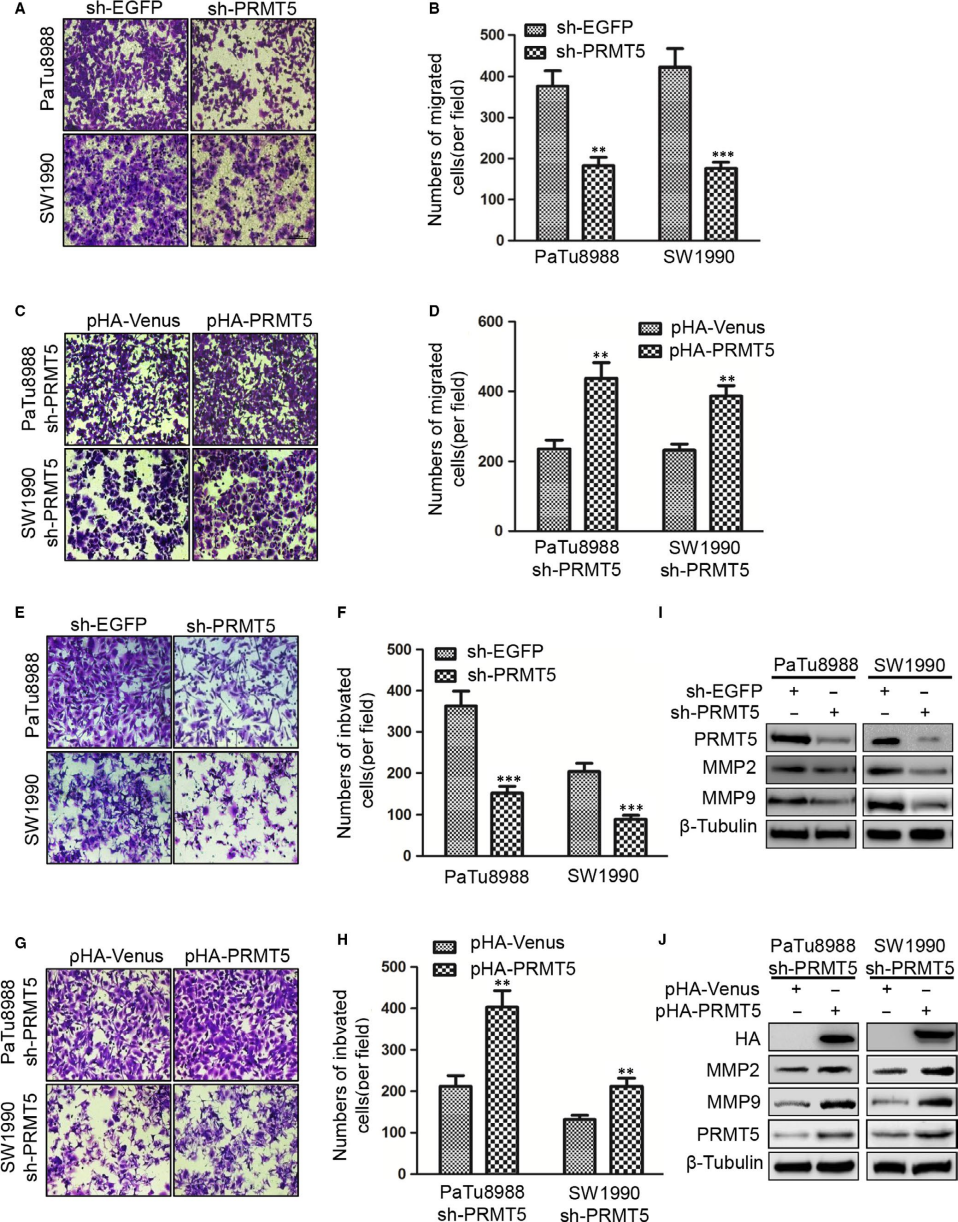
PRMT5 promotes cell migration and invasion in pancreatic cancer cells. A‐B, The migration changes were measured by transwell assay in PaTu8988 and SW1990 sh‐PRMT5 stable infected cells (Student's *t* test: **P < .01, ***P < .001). C‐D, After transfected with pHA‐Venus or pHA‐PRMT5 plasmid in PaTu8988 and SW1990 sh‐PRMT5 stable infected cells, the migration changes were measured by transwell assay (Student's *t* test: **P < .01). E‐F, The invasion changes were measured by transwell assay in PaTu8988 and SW1990 sh‐PRMT5 stable infected cells (Student's *t* test: ***P < .001). The number of cells passing through the reconstituted basement membrane was 363.13 ± 26.25 and 152.54 ± 9.13 in sh‐EGFP and sh‐PRMT5 PaTu8988 cells, and 204.38 ± 18.38 and 89.54 ± 6.75 in sh‐EGFP and sh‐PRMT5 SW1990 cells, respectively. G‐H, After transfected with pHA‐Venus or pHA‐PRMT5 plasmid in PaTu8988 and SW1990 sh‐PRMT5 stable infected cells, the invasion changes were measured by transwell assay (Student's *t* test: **P < .01). Numbers of cells passing through the reconstituted basement membrane were 212.33 ± 12.54 and 403.13 ± 35.42 in PaTu8988 cells, 132.04 ± 8.29 and 212.21 ± 16.46 in SW1990 cells, respectively. I‐J, The protein levels of MMP2 and MMP9 were measured by Western blot

Furthermore, the effect of PRMT5 on pancreatic cancer cell migration was determined by transwell migration assay. The cell number of passing through the transwell chambers was used as an index to evaluate the migration ability of PaTu8988 and SW1990 cells. The numbers of cells passing through the transwell chambers were 377.25 ± 22.29 and 182.50 ± 10.63 in sh‐EGFP and sh‐PRMT5 PaTu8988 cells, and 421.86 ± 29.36 and 176.13 ± 8.46 in sh‐EGFP and sh‐PRMT5 SW1990 cells, respectively (Figure 3A‐B). These results indicated that PRMT5 knockdown significantly inhibited the migration of pancreatic cancer cells. To confirm the above results, the sh‐PRMT5 PaTu8988 cells or SW1990 cells were transfected with pHA‐Venus or pHA‐PRMT5 plasmids, respectively. We found that the number of cells passing through the transwell chambers was 236.38 ± 13.79 and 436.50 ± 35.63 in PaTu8988 cells, 232.42 ± 13.25 and 385.75 ± 29.54 in SW1990 cells, respectively (Figure 3C‐D), indicating that ectopic PRMT5 re‐expression could rescue the effect of knockdown‐mediated inhibition on cell migration. It is suggested that PRMT5 performs a very important function on the cell migration in pancreatic cancer cells.

### 3.4 PRMT5 promotes cell invasion in pancreatic cancer cells

Then, we examined the effect of PRMT5 on the invasive abilities of the pancreatic cancer cells performed with transwell invasion assay. The cell number of passing through the reconstituted basement membrane was used as an index to evaluate the invasive ability of PaTu8988 and SW1990 cells. The number of cells passing through the reconstituted basement membrane in sh‐EGFP was less than in sh‐PRMT5 SW1990 and PaTu8988 cells, indicated that PRMT5 knockdown significantly inhibited the invasion of pancreatic cancer cells (Figure 3E‐F). To confirm the above results, the sh‐PRMT5 PaTu8988 cells or SW1990 cells were transfected with pHA‐Venus or pHA‐PRMT5 plasmids, respectively. We found that the number of cells passing through the reconstituted basement membrane was contrary to the above, indicating that ectopic PRMT5 re‐expression could rescue the effect of knockdown‐mediated inhibition on cell invasion(Figure 3G‐H). To better understand the molecules involved in PRMT5 signalling‐induced pancreatic cancer cells invasion, we identified whether PRMT5 affected MMP2 and MMP9 expression in pancreatic cancer cells. We found that knockdown of PRMT5 reduced the protein levels of MMP‐2 and MMP‐9 (Figure 3I), and ectopic PRMT5 re‐expression reversed the protein levels of MMP‐2 and MMP‐9 (Figure 3J). It is suggested that PRMT5 plays a major role on the cell invasion in pancreatic cancer cells.

### 3.5 PRMT5 promotes EMT via activating EGFR/AKT/β‐catenin signalling in pancreatic cancer cells

**Figure 4:**
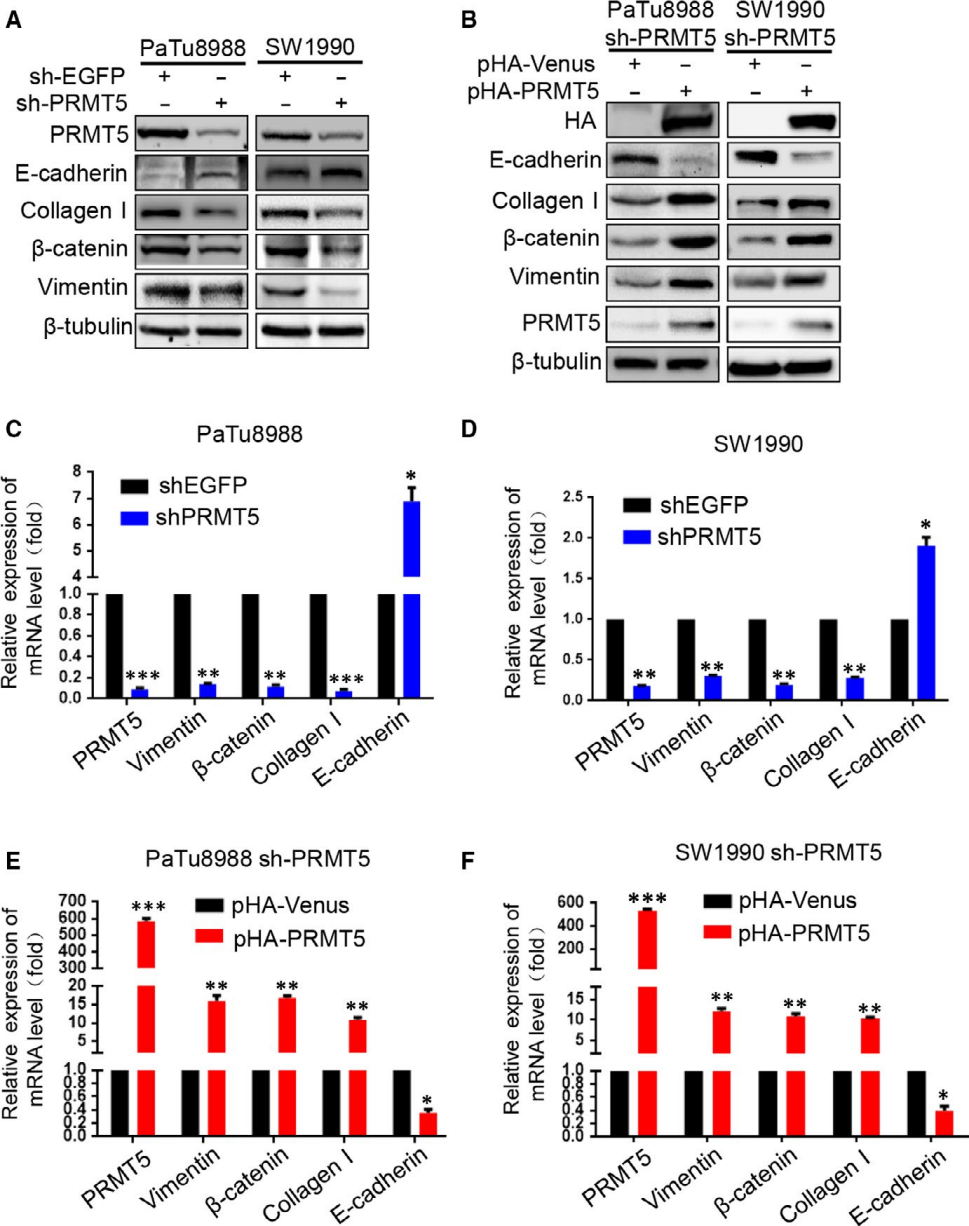
PRMT5 promotes EMT in pancreatic cancer cells. A, The protein levels of E‐cadherin, collagen I, β‐catenin and Vimentin were measured by Western blot in PaTu8988 and SW1990 sh‐PRMT5 stable infected cells. B, After transfected with pHA‐Venus or pHA‐PRMT5 plasmid in PaTu8988 and SW1990 sh‐PRMT5 stable infected cells, the protein levels of E‐cadherin, collagen I, β‐catenin and Vimentin were measured by Western blot. C‐D, Vimentin, β‐catenin and Collagen I mRNA levels were reduced while E‐cadherin mRNA level was increased in shPRMT5‐PaTu8988 and SW1990 cells. *P < .05, **P < .01, ***P < .001. E‐F, Vimentin, β‐catenin and Collagen I mRNA levels were increased while E‐cadherin mRNA level was reduced in sh‐PRMT5 PaTu8988 and SW1990 cells transfected with pHA‐PRMT5 plasmid. *P < .05, **P < .01, ***P < .001

**Figure 5:**
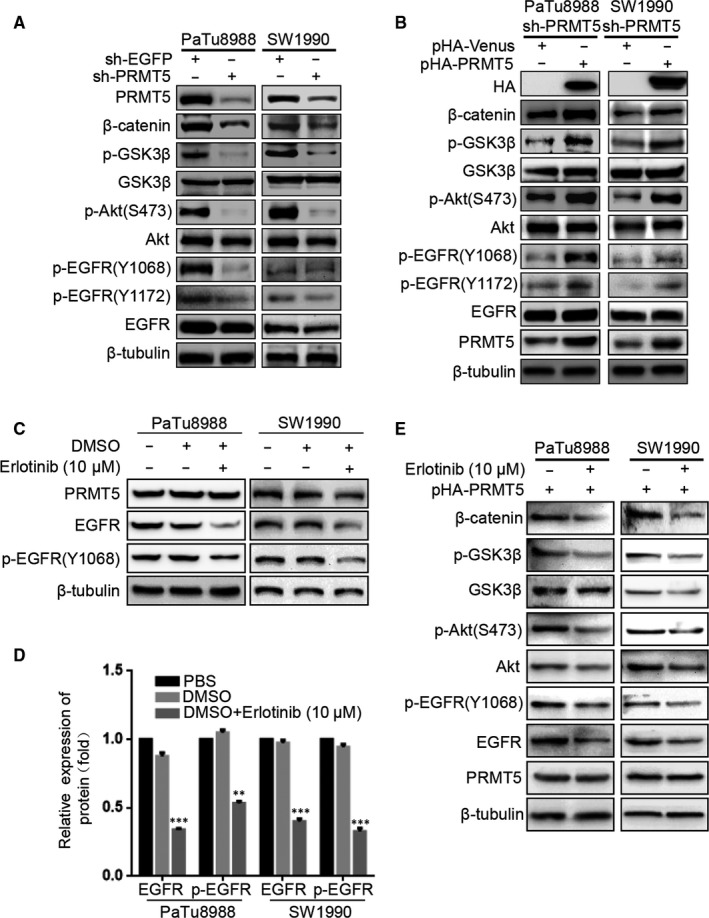
PRMT5 activates EGFR/AKT/β‐catenin signalling in pancreatic cancer cells. A, Wnt/β‐catenin and EGFR signalling relative proteins were detected by Western blot in PaTu8988 and SW1990 sh‐PRMT5 stable infected cells. B, After transfected with pHA‐Venus or pHA‐PRMT5 plasmid in PaTu8988 and SW1990 sh‐PRMT5 stable infected cells, EGFR/AKT/β‐catenin signalling relative proteins were detected by Western blot. C, Pancreatic cancer cells were treated with EGFR inhibitor at 0, 10 μmol/L for 3 d. The expression of EGFR, p‐EGFR (Y1068) and PRMT5 was determined by Western blotting. D, The quantification of EGFR and p‐EGFR is shown. **P < .01, ***P < .001. E, Wnt/β‐catenin and EGFR signalling relative proteins were detected by Western blot in PaTu8988 and SW1990 pHA‐PRMT5 stable infected cells treated with Erlotinib (10 μmol/L) for 3 d

To probe the molecular basis for PRMT5‐enhanced cell motility, we next examined some EMT biomarkers such as E‐cadherin, collagen I, β‐catenin and Vimentin. Both at mRNA and protein levels, silencing PRMT5 induces epithelial marker E‐cadherin expression and down‐regulates expression of mesenchymal markers including Vimentin, collagen I and β‐catenin in PaTu8988 and SW1990 cells, whereas ectopic PRMT5 re‐expression partially reverses these changes (Figure 4A‐F). The above results indicated that PRMT5 promoted pancreatic cancer proliferation, invasion, migration and EMT. To investigate the possible mechanism, we tested the effect of PRMT5 knockdown on invasion‐related signalling (Figure 5A‐B). We found that PRMT5 knockdown decreased the phosphorylation level of AKT, as well as its downstream p‐GSK‐3β, and then resulted in β‐catenin down‐regulation. Expectedly, ectopic PRMT5 re‐expression reversed these changes. Previous study proved that EGFR is methylated by an arginine methyltransferase PRMT5.^17^ Considering EGFR as the upstream signalling of AKT pathway, we speculate that EGFR signalling also regulates PRMT5‐induced EMT in pancreatic cancer cells. So, we utilized the Western blot to detect the level of EGFR, p‐EGFR (Y1068) and p‐EGFR (Y1172). As observed in Figure 5A‐B, PRMT5 knockdown decreased the phosphorylation level of EGFR (at Y1068 and Y1172) in pancreatic cancer cells, while ectopic PRMT5 re‐expression reversed these changes. Additionally, we found that the expression of EGFR, p‐EGFR(Y1068), Akt, p‐Akt(S473), GSK3β, p‐GSK3β and β‐catenin was decreased in PaTu8988 and SW1990 pHA‐PRMT5 stable infected cells treated with Erlotinib (10 μmol/L) (Figure 5C‐E, Figure S1D‐E). It is suggested that inhibitors of EGFR/AKT/β‐catenin signalling had influence on the effect of PRMT5 and the function of PRMT5 on the EGFR/AKT/β‐catenin signalling. Thus, these data strongly suggest that PRMT5 regulates EGFR/AKT/β‐catenin signalling, which probably contributes to PRMT5‐induced EMT in pancreatic cancer cells.

## 4 DISCUSSION

In this study, we confirm that PRMT5 is overexpressed in human pancreatic cancer at both mRNA and protein levels, and acts as an independent prognostic factor for patient outcome. Furthermore, we find that PRMT5 promotes EMT via stimulating EGFR/AKT/β‐catenin pathway for the first time. All these findings suggest that PRMT5 may function as an oncogene and be a candidate for diagnosis and prognosis in pancreatic cancer.

Previous studies have determined that PRMT5 may function as an oncogene to promote cancer cell growth.^18^, ^19^ For example, Li Z et al^20^ found that PRMT5 promoted cell proliferation, tumorigenicity, tumour invasion and metastasis as LINC01138 acted as an oncogenic driver. Deng X et al^11^ found that PRMT5 promotes prostate cancer cell growth by epigenetically activating transcription of the androgen receptor (AR) in prostate cancer cells. Especially in lung cancer cells, PRMT5 was proved to promote cell proliferation through direct interaction with Akt and regulation of Akt activity.^21^ More recently, PRMT5 was demonstrated that lead to FBW7 expression to promote tumorigenesis in pancreatic cancer.^14^ In this study, we find that PRMT5 promotes cell proliferation, colony formation, migration and invasion in pancreatic cancer cells, and promotes tumorigenesis. EMT has been associated with various tumour functions, including tumour initiation, malignant progression and tumour cell migration. More importantly, we confirm that PRMT5 promotes EMT through EGFR/AKT/β‐catenin pathway in pancreatic cancer cells. Collectively, these data indicate that PRMT5 may function as an oncogene and is a key mediator in carcinogenesis and progression of pancreatic cancer.

It is well‐established that β‐catenin is dependent on Wnt signalling to promote cancer progression in various tumours. Upon canonical Wnt‐signal, Wnt receptors inhibit the β‐catenin phosphorylation, and facilitate β‐catenin stabilization and β‐catenin translocation into the nuclei.^22^ Recently, Stephanie Grainger et al^23^ found that EGFR‐mediated phosphorylation of Fzd9b via β‐catenin‐dependent Wnt signalling to regulate haematopoietic stem and progenitor cell emergence, indicating that EGFR activation is required as a cofactor for β‐catenin‐dependent Wnt signalling. Also, it is found that β‐catenin activation is independent of canonical Wnt signalling. For example, calreticulin increased β‐catenin protein expression to promote EMT via Integrin/EGFR‐ERK/MAPK signalling,^24^ and EGF‐induced nuclear translocation of SHCBP1 directly increased acetylation of β‐catenin to enhanced NSCLC cellular stemness,^25^ which is suggested that EGFR signalling can drive β‐catenin activation via various routes. In this study, we provide a novel notion that PRMT5 induces the phosphorylation of EGFR, and then activates phosphorylation of Akt and its downstream GSK3β, and thereby up‐regulates expression of β‐catenin to enhance the migratory and invasive motility and promotes EMT of pancreatic cancer cells. Moreover, previous study proved that PRMT5‐mediated EGFR Arg1175 methylation positively modulates EGF‐induced EGFR trans‐autophosphorylation at Tyr 1173, but have no effect on EGFR trans‐autophosphorylation at Tyr 1086, 845, 992, 1045 and 1148.^17^ Herein, we for the first time find that PRMT5 promotes the autophosphorylation of EGFR at Y1068 and Y1172 to activate Akt‐β‐catenin axis in pancreatic cancer cells.

In summary, our study provides proof that PRMT5 promotes EMT in pancreatic cancer cells probably through activating EGFR/AKT/β‐catenin signalling. And a noteworthy feature of our study is that we find the expression of p‐EGFR (Y1068) and p‐EGFR (Y1172) is increased remarkably. Above all, we for the first time established the link between PRMT5 and EGFR/AKT/β‐catenin signalling in pancreatic cancer. All these findings suggest that PRMT5 is a potential biomarker for diagnostics and prognosis of pancreas cancer.

## CONFLICT OF INTEREST

The authors confirm that there are no conflicts of interest.

## AUTHORS’ CONTRIBUTIONS

Lu Ge and Huizhi Wang performed most experiments and wrote the manuscript; Xiao Xu performed some experiments and candled data from database; Junbo He performed some experiments; Wanxin Peng, Fengyi Du, Youli Zhang, Min Xu and Aihua Gong designed experiments and organized the manuscript.

## Data Availability

The data that support the findings of this study are available from the corresponding author upon reasonable request.
