# Supplementary figures and images for: PRMT5 promotes epithelial‐mesenchymal transition via EGFR‐β‐catenin axis in pancreatic cancer cells
Source: J Cell Mol Med. 2019 Dec 18;24(2):1969–79. doi: 10.1111/jcmm.14894 (PMC6991680; doi:10.1111/jcmm.14894)

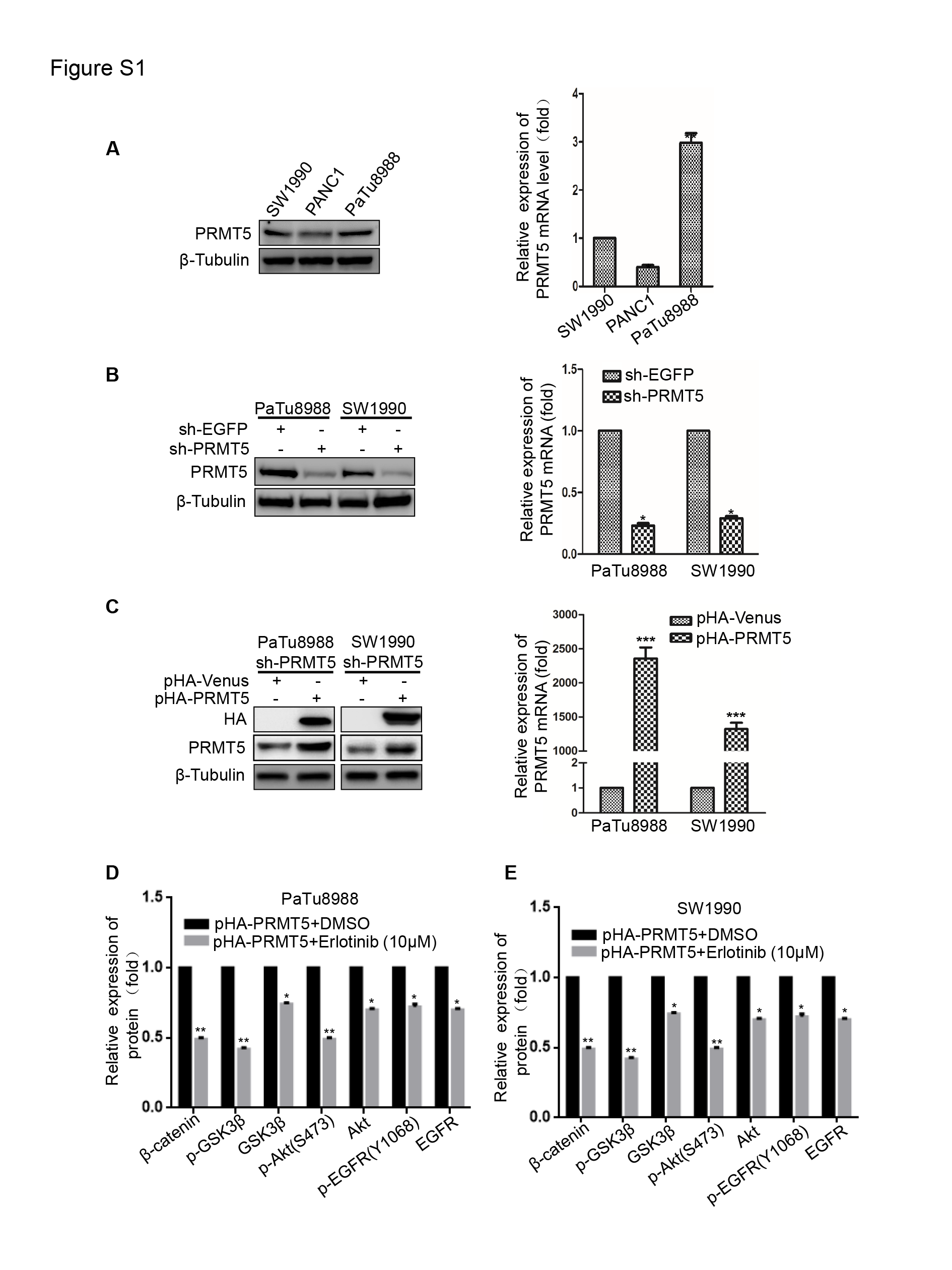

Supplement: Supplementary file 1 [file JCMM-24-1969-s001.tif]
